# *Cloacibacillus porcorum* sp. nov., a mucin-degrading bacterium from the swine intestinal tract and emended description of the genus *Cloacibacillus*

**DOI:** 10.1099/ijs.0.044719-0

**Published:** 2013-06

**Authors:** T. Looft, U. Y. Levine, T. B. Stanton

**Affiliations:** Food Safety and Enteric Pathogens Research Unit, National Animal Disease Center, Agricultural Research Service, United States Department of Agriculture, Ames, IA 50010, USA

## Abstract

A novel anaerobic, mesophilic, amino-acid-fermenting bacterium, designated strain CL-84^T^, was isolated from the swine intestinal tract on mucin-based media. Cells were curved rods (0.8–1.2×3.5–5.0 µm), stained Gram-negative and were non-motile with no evidence of spores. Strain CL-84^T^ produced acetate, propionate, formate and butyrate as the end products of metabolism when grown on serine. Optimum growth occurred at 39 °C and pH 6.5. The major cellular fatty acids were iso-C_15 : 0_, iso-C_15 : 0_ 3-OH, iso-C_17 : 0_ and C_16 : 0_, distinguishing strain CL-84^T^ from closely related species. The DNA G+C content of strain CL-84^T^ was 55.1 mol%. 16S rRNA gene sequence analysis showed that strain CL-84^T^ shared 90–95 % similarity with characterized genera within the phylum *Synergistetes*, family *Synergistaceae**.* Phylogenetic analysis showed that strain CL-84^T^ was related to, but distinct from, *Cloacibacillus evryensis**.* Based on these findings, we propose that strain CL-84^T^ represents a novel species of the genus *Cloacibacillus*. We further propose the name *Cloacibacillus porcorum* sp. nov. be designated for this species. The type strain is CL-84^T^ ( = DSM 25858^T^ = CCUG 62631^T^). An emended description of the genus *Cloacibacillus* is provided.

Members of the recently described phylum *Synergistetes* have been identified in a diverse range of anaerobic environments, including anaerobic digesters (Ganesan *et al.*, 2008) and some infections in the human body (e.g. peritoneal fluid, soft tissues, blood and periodontal pockets) (Vartoukian *et al.*, 2007). Despite their culture-independent identification in a wide range of environments, there are few cultured representatives of this phylum. *Synergistes jonesii*, the first characterized *Synergistetes* species, was isolated from a goat rumen. *S. jonesii* degrades toxic pyridinediols in the animals’ diet, and in turn the animal’s gut provides required nutrients (Allison *et al.*, 1992). In this paper we describe the isolation and characterization of a mucin-degrading bacterium, strain CL-84^T^, from the swine intestine. We suggest that strain CL-84^T^ represents a novel species of the genus *Cloacibacillus*, for which the name *Cloacibacillus porcorum* sp. nov. is proposed. How many taxa may share this trait is unclear, but to our knowledge, this is the first description of a member of the phylum *Synergistetes* that can use mucin as a sole source of carbon and energy.

## 

Strain CL-84^T^ was one of eight *Synergistetes* strains isolated during the characterization of mucosa-associated and mucin-degrading micro-organisms from the swine intestinal tract. The gently rinsed mucosal surface of a pig caecum was scraped with a sterile microscope slide and inoculated into minimal medium containing mucin. A series of three enrichments (10 days each) in broth, containing a basal medium, described below, and 1 % (w/v) hog gastric mucin (HGM) (Sigma-Aldrich), were used to enhance the growth of mucolytic bacteria before inoculation on solid media. Mucin-degrading bacteria were isolated on solid basal medium supplemented with 1 % (w/v) HGM after incubation at 39 °C for 5 days. Pure cultures were obtained after isolates were streaked for isolation three times. All cultures were inoculated and incubated (39 °C) in a Coy anaerobic chamber inflated with an atmosphere of N_2_ (85 %), CO_2_ (5 %) and H_2_ (10 %). The basal medium contained (per litre) 0.45 g CaCl_2_, 0.45 g MgSO_4_, 2.25 g KH_2_PO_4_, 2.25 g K_2_HPO_4_, 4.5 g NaCl, 4.5 g (NH_4_)_2_SO_4_, 0.05 % cysteine, 0.05 g haemin, 0.0001 % resazurin and 1.6 % Noble agar.

## 

Strain CL-84^T^ grew optimally on brain heart infusion broth with 0.05 % cysteine and 0.0001 % resazurin, and supplemented with 20 mM arginine and histidine (BHIAH). This medium was used to maintain cultures. After 3 days growth at 39 °C on BHIAH medium, strain CL-84^T^ reached a terminal OD_620_ of 1.2, representing 1.5×10^9^ c.f.u. ml^−1^. The calculated doubling time was 8 h. Cells of strain CL-84^T^ cultured in BHIAH broth had a curved-rod shape, were non-motile (determined with motility test medium; Greene *et al.*, 1951) and spores were not seen (Fig. 1a). On BHIAH plates after 5 days growth, strain CL-84^T^ produced small semi-translucent brown colonies that were 1 mm in diameter. Gram strain was negative. Ultrathin sections were prepared for transmission electron microscopy (TEM) from 4-day-old cultures, stained with uranyl acetate and lead citrate, and examined with a Tecnai 12 G^2^ Biotwin microscope (FEI). Micrographs revealed a cell outer envelope structure consistent with Gram-negative cells with a thin peptidoglycan layer surrounded by an outer membrane (Fig. 1b). No spores or inclusion bodies were seen. Cells were variable in size (0.8–1.2 µm × 3.5–5.0 µm). The TEM appearance and Gram staining characteristics were consistent with the Gram-negative envelope characteristics of other *Synergistetes* spp.

**Fig. 1.**
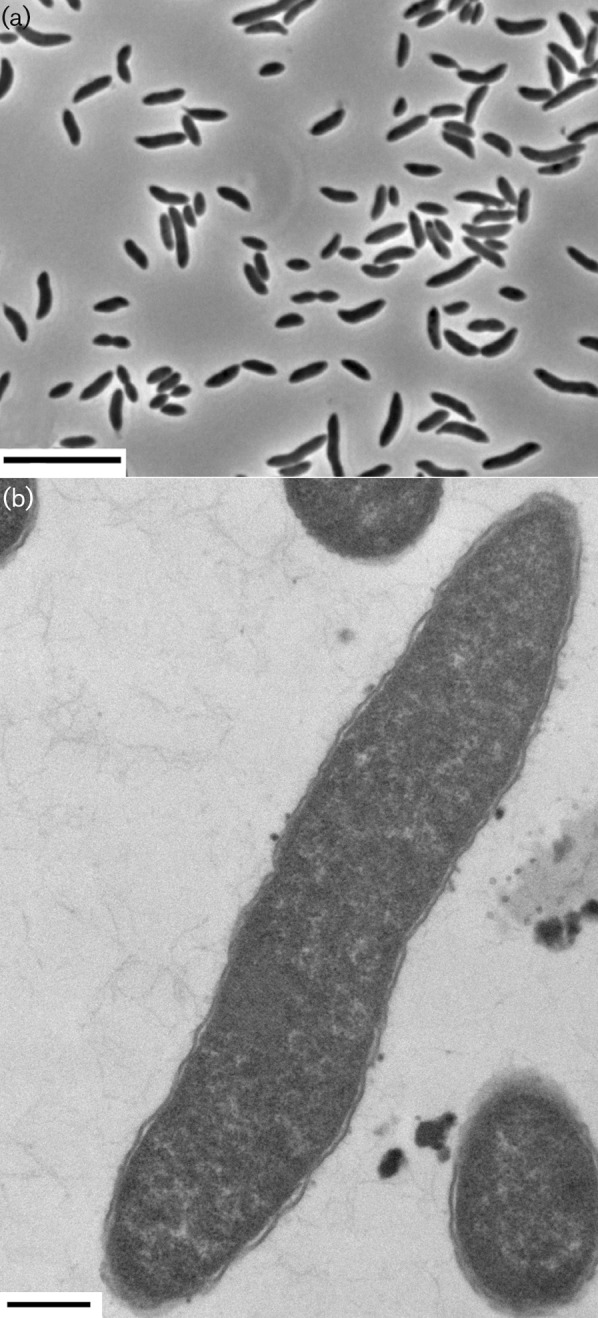
(a) Phase-contrast micrograph of cells of strain CL-84^T^** grown in BHIAH medium. Bar, 10 µm. (b) Transmission electron micrograph of ultrathin sections of cells of strain CL-84^T^, showing a loose outer cell membrane consistent with a Gram-negative outer cell envelope and a negative Gram-staining reaction. Bar, 500 nm.

## 

Genomic DNA was extracted from pelleted cells of strain CL-84^T^ by following the protocol of Dale & Greenaway (1985). PCR amplification was carried out as described by Downes *et al.* (2005), with the conserved bacterial primers 8F (Wilmotte *et al.*, 1993) and 1492R (Stackebrandt & Goodfellow, 1991). Purified PCR products were sequenced, yielding nearly full-length (1447 bp) sequences for the 16S rRNA gene of strain CL-84^T^. Taxonomic assessments of the 16S rRNA gene sequences were made using the Ribosomal Database project (RDP) web tools (http://rdp.cme.msu.edu/) (Cole *et al.*, 2009), which placed strain CL-84^T^** within the phylum *Synergistetes*. The closest matches were to *Cloacibacillus evryensis* 158^T^ (95 % sequence similarity) and *S. jonesii* 78-1^T^ (90 %). *C. evryensis* was isolated from a municipal anaerobic waste digester (Ganesan *et al.*, 2008) and *S. jonesii* was isolated from a goat rumen (Allison *et al.*, 1992). Support for other *Synergistetes* bacteria being associated with mucus and possibly utilizing mucin can be found from their identification from subgingival plaque samples, and optimized growth (in co-culture) by the addition of mucin to the media (Vartoukian *et al.*, 2010).

Neighbour-joining, maximum-parsimony and maximum-likelihood phylogenetic analyses were performed based on the alignment of the 16S rRNA gene sequence from strain CL-84^T^** with Silva’s SINA web aligner (Pruesse *et al.*, 2007) in the software package arb (Ludwig *et al.*, 2004). The methods used to construct the tree were arb neighbour-joining (10 000 bootstrap replicates), maximum-parsimony with dnapars v3.6 (1000 bootstraps) and maximum-likelihood with RAxML (‘advanced bootstrap+refinement of BS tree’ algorithm, GTRGAMMA model, 1000 bootstraps). All three analyses produced trees with the same topology, and therefore only the neighbour-joining tree is presented (Fig. 2). Strain CL-84^T^** grouped with uncharacterized isolates from infected human blood (GenBank accession numbers GQ258969, EF551162 and EF551160) (Marchandin *et al.*, 2010) and peritoneal fluid samples (DQ412721) (Horz *et al.*, 2006). These sequences were approximately 99 % similar to each other and to that of strain CL-84^T^, forming a cluster previously designated OTU cluster 2 by Ganesan *et al.* (2008). In contrast, the 16S rRNA gene sequences from *C. evryensis* 158^T^ and *S. jonesii* 78-1^T^ were only 95 and 90 % similar to the sequences in OTU cluster 2, respectively. Strain CL-84^T^ and related sequences formed a branch distinct from *C. evryensis* (OTU cluster 1), within the genus *Cloacibacillus*.

**Fig. 2.**
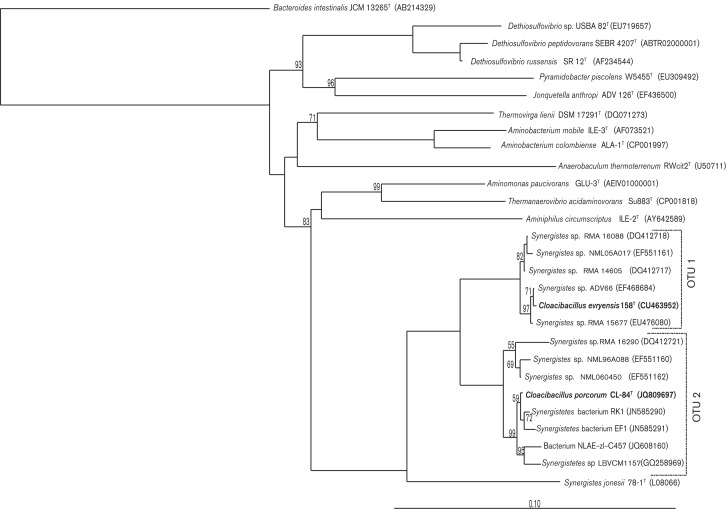
Neighbour-joining phylogentic tree of the proposed species *Cloacibacillus porcorum* sp. nov. (strain CL-84^T^) and selected reference type strains based on partial 16S rRNA gene sequences (all ≥1334 bp). The tree is rooted by *Bacteroides intestinalis* JCM 13265^T^. Numbers by the branches of the tree represent percentage bootstrap values of 10 000 resamplings, and are only noted if the percentage was greater than 50 %. OTU 1 and 2, previously described by Ganesan *et al.* (2008), are shown and represent a 16S sequence divergence of ~5 %. Bar, 10 nt substitutions per 100 nt.

As part of a larger survey of swine intestinal microbes, the genome of strain CL-84^T^** was sequenced using a 454 titanium pyrosequencing platform (454 Life Sciences). The gyrase B gene sequence of strain CL-84^T^ (GenBank accession no. JX443487) was obtained from the genome sequence and compared with that of *C. evryensis*, obtained from the finished genome sequence (IMG/GEBA database: 6858). The gyrase B nucleotide sequence from strain CL-84^T^** showed only 90 % similarity to *C. evryensis*, which supports the conclusion that these are different species. Sequence analysis revealed that strain CL-84^T^** has a DNA G+C content of 55.1 mol%.

## 

Mucins are glycoproteins with carbohydrate side chains connected to a protein backbone by *O*-glycosidic links (Nataro, 2005) and are the major component of mucus. The carbohydrate side groups are made up of the sugars galactose, fucose, *N*-acetyl-d-galactosamine, *N*-acetyl-d-glucosamine, sialic acid and mannose (Allen *et al.*, 1998). Mucolytic bacteria use proteases and glycosidases to degrade host mucin at polypeptide and glycosidic bonds, respectively (Bradshaw *et al.*, 1994). Growth on different mucin components was examined by preparing basal medium containing 0.2 % (w/v) yeast extract with 0.5 % (w/v) of each of the following: chondroitin sulfate sodium salt, *N*-acetyl-d-glucosamine, *N*-acetyl-d-galactosamine, hyaluronan biotin sodium salt, mannose, *N*-acetylneuraminic acid, d-galactose or l-fucose. Strain CL-84^T^** was further evaluated for its ability to degrade mucin *O*-linked glycans, purified from the HGM, as described by Martens *et al.* (2008). Growth on a variety of amino acids was examined by supplementing basal medium containing 0.2 % (w/v) yeast extract with 10 mM of each of the following: arginine, histidine, lysine, serine, tryptophan, alanine, glutamate, aspartate, proline, glycine, cysteine, phenylalanine, isoleucine, leucine, valine, threonine, methionine, glutamine, asparagine or tyrosine. Strain CL-84^T^** grew on BHI and Casamino acids broth (both used for growth of *C. evryensis*), but grew faster on BHIAH. Growth was observed on a limited number of amino acids and whole mucin and mucin components. Growth on whole mucin and mucin *O*-linked glycans was also tested and observed with *C. evryensis* (OD_620_ of 0.8 and 0.06, respectively). Growth results for strain CL-84^T^** are summarized in Table 1 as OD_620_ values.

**Table 1.** Fermentation products from substrates that support growth of strain CL-84^T^ in basal medium No growth was observed on methionine, aspartate, valine, proline, isoleucine, lysine, alanine, phenylalanine, leucine, glucose, glutamine, glycine, asparagine, glutamate or tyrosine.

Substrate	Formic acid*	Acetic acid*	Propionic acid*	Butyric acid*	OD_620_†
BHIAH	−	++	+	−	+++
Histidine	+	+	+	−	++
Arginine	+	+	+	−	++
Tryptophan	−	+	−	−	+
Cysteine	+	++	+	−	++
Threonine	−	+	−	−	+
Serine	+	+	−	+	+
Serine, threonine, proline	+	++	+	+	++
(−)-*N*-Acetylneuraminic acid	+	−	+	−	+
Hyaluronan biotin sodium salt	+	+	+	−	++
Mannose	+	+	−	−	++
Chondroitin sulfate sodium salt	+	+	+	−	+
Fucose	−	+	+	−	+
d-Galactose	−	+	−	−	+
*N*-Acetyl-d-galactosamine	−	−	+	−	+
*N*-Acetyl-d-glucosamine	−	−	−	−	+
Mucin	+	+	+	−	+
Mucin *O*-linked glycans	+	++	+	−	+

* Measured by GC. All values were corrected for the small amount of short-chain fatty acids formed in the control tubes. Experiments were conducted in triplicate and mean values are given. −, <1.0 mM; +, 1.0–5.0 mM; ++, 5.1–10.0 mM.

† OD_620_ values: +, 0.05–0.1; ++, 0.2–0.5; +++, 0.6–1.2.

## 

The supernatants from cultures on each substrate were analysed for fermentation acids by GC of butyl esters (Salanitro & Muirhead, 1975; Stanton & Lebo, 1988). Major fermentation products were formate, acetate or propionate; however, growth on serine, both alone and with threonine and proline, also produced buyrate (Table 1). Strain CL-84^T^ contained butyryl-CoA : acetate CoA-transferase (E.C. 2.8.3.8) activity [specific activity = 9.88±0.45 µmol min^−1^ (mg protein)^−1^] as determined using cells cultured on basal medium with serine. The assay for transferase activity was performed on French press extracts of strain CL-84^T^ according to Buckel *et al.* (1981) except that it was evaluated at 412 nm, 39 °C, with 0.1 mM butyryl-CoA and 0.5 units (8.8 nkat) of citrate synthase. The most prevalent amino acids in mucin are serine and threonine (Schrager, 1970).** The ability of strain CL-84^T^ to produce butyrate when grown on serine may reflect its role in the gut, as butyrate has been shown to stimulate mucin synthesis in human colonic cell lines and animal mucosa (Finnie *et al.*, 1995; Hatayama *et al.*, 2007).

Additional biochemical characterizations were performed using Rapid ID 32 A and API ZYM test strips (bioMérieux). Strain CL-84^T^** showed positive arginine dihydrolase and glutamic acid decarboxylase activities and weak alkaline phosphatase and proline arylamidase activities. Additionally, the API ZYM test showed that strain CL-84^T^** had acid phosphatase, naphthol-AS-BI-phosphohydrolase, alkaline phosphatase, esterase (C4), esterase lipase (C8) and leucine arylamidase activities. Strain CL-84^T^** tested negative for catalase.

## 

Heat, pH and oxygen tolerance were tested in BHIAH medium. The pH range for growth of strain CL-84^T^** was pH 4–8, with optimal growth (reaching an OD_620_ of >1.0) at pH 6–7. Growth between 20 and 45 °C was observed. No viable cells remained after heating cultures to 80 °C for 30 min. Growth of CL-84^T^** was inhibited by NaCl concentrations above 1.4 % (w/v), with optimal growth at 0.6–0.8 % (w/v) NaCl. Strain CL-84^T^ cells were aerotolerant and still viable after 24 h of exposure to oxygen. Growth of CL-84^T^** was not observed on BHIAH plates, exposed to 1.0 % oxygen, after 1 week of incubation.

## 

The closest related type strain to CL-84^T^, *C. evryensis* 158^T^, was obtained from the DSM culture collection (accession no.19522). Both strain CL-84^T^** and *C. evryensis* 158^T^ were grown in 10 ml BHIAH medium to an OD_620_ of 1.0, and cell pellets were used for cellular fatty acid analysis. The cellular fatty acid composition was determined by Microbial ID Inc. with the GC-based MIDI Sherlock Microbial Identification System. The major cellular fatty acids of strain CL-84^T^ were iso-C_15 : 0_ (27.1 %), iso-C_15 : 0_ 3-OH (15.0 %), iso-C_17 : 0_ (11.7 %) and C_16 : 0_ (9.7 %). The profile for strain CL-84^T^ differed from that of *C. evryensis* 158^T^ in both the fatty acid types and the proportions of each component. The top three fatty acids for strain CL-84^T^** were iso-C_15 : 0_, iso-C_15 : 0_ 3-OH and iso-C_17 : 0_ (27.1, 15 and 11.7 %, respectively), while the top three for *C. evryensis* 158^T^ were iso-C_15 : 0_, iso-C_15 : 0_ 3-OH and C_17 : 1_ω11*c* (14.2, 14 and 12.4 %, respectively) (Table 2).

**Table 2.** Cellular fatty acid profiles (%) of strain CL-84^T^ and *C. evryensis* 178^T^, grown on BHIAH medium Taxa: 1, CL-84^T^; 2, *C. evryensis* 178^T^.

Fatty acid	Proportion of total fatty acids (%)	
	**1**	**2**
iso-C_15 : 0_	27.1	14.2
iso-C_15 : 0_ 3-OH	15	14
iso-C_17 : 0_	11.7	2.4
C_16 : 0_	9.7	1.8
iso-C_13 : 0_	7.6	5.8
C_17 : 0_	4.2	12
iso-C_17 : 1_ at 10	3.9	2.7
C_16 : 1_ω7*c*	3.7	1.6
C_18 : 0_	2.8	0
C_18 : 1_ω11*c*	2.6	0
C_17 : 1_ω11*c*	2.3	12.4
C_15 : 0_	1.9	4.8
C_16 : 1_ω11*c*	1.7	0
iso-C_17 : 1_ at 9	1.6	0
C_13 : 0_	0	3.4
C_15 : 0_ 3-OH	0	7.7
C_15 : 1_ω6*c*	0	4.3
C_16 : 1_ω5*c*	0	1.8
C_17 : 1_ω3*c*	0	1.3
C_17 : 1_ω6*c*	0	5.3
Summed features*		
C_14 : 0_ 3-OH/iso-C_16 : 1_	3.2	2.2
C_17 : 0_ DMA/unknown at 17.49†	0	1.8

* Summed features represent two fatty acids that cannot be separated with the MIDI system.

† Unknown fatty acids do not have a name listed in the MIDI system library; values are equivalent chain lengths.

## 

Antibiotic minimum inhibitory concentration (MIC) assays were modified from Allen *et al.* (2009) by incubating anaerobically in BHIAH for 1 week. Strain CL-84^T^** was susceptible to tylosin, lincomycin, chlortetracycline, penicillin, florphenicol, ceftiofur and carbadox (MIC <4 µg µl^−1^) and resistant to vancomycin and sulfathiazole (MIC 512 µg µl^–1^ for each). Other *Synergistetes* spp. have also been shown to be resistant to vancomycin (Allison *et al.*, 1992; Downes *et al.*, 2009; Ganesan *et al.*, 2008).

## Emended description of the genus *Cloacibacillus* Ganesan *et al*. 2008

The description of the genus *Cloacibacillus* follows that of Ganesan *et al.* (2008) with the following modifications and additions. The ability to grow on mucin and mucin components, as a sole carbon source, is a trait shared by members of this genus. Cells are glucose non-fermenters, but can grow on fucose. The predominant fatty acids include iso-C_15 : 0_ and iso-C_15 : 0_. Differential characteristics between strain CL-84^T^ and *C. evryensis* 158^T^ and *S. jonesii* 78-1^T^ are given in Table 3.

**Table 3.** Differential characteristics between strain CL-84^T^ and related species in the phylum *Synergistetes* Taxa: 1, CL-84^T^; 2, *C. evryensis* 158^T^; 3, *S. jonesii* 78-1^T^.

Characteristic	1	2*	3†
Isolation source	Swine intestinal tract	Anaerobic digester	Goat rumen
Cell morphology	Slightly curved rods	Straight rods	Rods
Cell size (µm)	0.8–1.2×3.5–5.0	0.8–1.0×2.0–3.0	0.6–0.8×1.2–1.8
16S rRNA similarity to CL-84^T^ (%)	100	95	90
Optimum growth temperature (°C)	39	35–40	39
DNA G+C content (mol%)	55.1	55.8	58
Fermentation	Proteolytic, some carbohydrates	Proteolytic	Proteolytic
Short-chain fatty acids produced during fermentation	Acetate, propionate, formate; butyrate, only when serine is supplied	Acetate, propionate, butyrate, valerate	Acetate, propionate; formate only when histidine is supplied
Major cellular fatty acids	iso-C_15 : 0_, iso-C_15 : 0_ 3-OH, iso-C_17 : 0_, C_16 : 0_	iso-C_15 : 0_, iso-C_15 : 0_ 3-OH, C_17 : 1_ω11*c*, C_17 : 0_	C_17 : 0_, C_20_ cyclo, C_17 : 1_ω6*c*, C_15 : 0_
Additional features	Degrades mucin	Degrades mucin	Degrades 3,4-dihydroxy pyridine

* Data from Ganesan *et al.* (2008).

† Data from Allison *et al.* (1992).

## Description of *Cloacibacillus porcorum* sp. nov.

*Cloacibacillus porcorum* (por.co′rum. L. n. *porcus* swine, pig; L. masc. pl. n. *porcorum* of/from pigs).

Cells are obligately anaerobic, non-motile and curved-rod shaped. Cells ferment amino acids, and can use mucin and mucin components as a sole carbon source. Fermentation products are acetate, propionate and formate but only butyrate is produced from serine. This bacterium is an intestinal commensal of swine. Cells have a Gram-negative cell-wall structure and range in size from 0.8 to 1.2 µm wide and 3.5 to 5.0 µm long. Strong growth is obtained in BHI medium, and growth is enhanced by the addition of histidine and arginine, but not glucose. After 7 days incubation, colonies are 1 mm in diameter, circular, shiny, brown and semi-translucent. The main cellular fatty acids are iso-C_15 : 0_, iso-C_15 : 0_ 3-OH, iso-C_17 : 0_ and C_16 : 0_. Cells are resistant to vancomycin and sulfathiazole but are susceptible to tylosin, lincomycin, chlortetracycline, penicillin, florphenicol, ceftiofur and carbadox. Optimal growth occurs at 39 °C and pH 6.5.

The type strain, CL-84^T^ ( = DSM 25858^T^ = CCUG 62631^T^), was isolated from the mucosal lining of a pig caecum in Ames, Iowa, USA. The DNA G+C content of the type strain is 55.1 mol%.
